# Investigating the relationship between haematological parameters and metabolic syndrome: A population‐based study

**DOI:** 10.1002/edm2.407

**Published:** 2023-01-27

**Authors:** Mohammad Javad Najafzadeh, Amir Baniasad, Reza Shahabinejad, Mahdieh Mashrooteh, Hamid Najafipour, Mohammad Hossein Gozashti

**Affiliations:** ^1^ Gastroenterology and Hepatology Research Center Institute of Basic and Clinical Physiology Sciences, Kerman University of Medical Sciences Kerman Iran; ^2^ Endocrinology and Metabolism Research Center Institute of Basic and Clinical Physiology Science, Kerman University of Medical Sciences Kerman Iran; ^3^ Physiology Research Center Institute of Basic and Clinical Physiology Sciences, Kerman University of Medical Sciences Kerman Iran; ^4^ Modeling in Health Research Center Institute for Futures Studies in Health, Kerman University of Medical Sciences Kerman Iran; ^5^ Cardiovascular Research Center Institute of Basic and Clinical Physiology Sciences, Kerman University of Medical Sciences Kerman Iran; ^6^ Endocrinologist, Endocrinology and Metabolism Research Center Institute of Basic and Clinical Physiology Sciences, Kerman University of Medical Sciences Kerman Iran

**Keywords:** complete blood count, Haematological parameters, leukocytes, metabolic syndrome, obesity

## Abstract

**Background:**

Metabolic syndrome (MetS) is a global public health concern. Chronic inflammation plays a role in MetS; haematological inflammatory parameters can be used as MetS predicting factors.

**Objective:**

Hereditary and environmental factors play an important role in the development of MetS. This study aimed to determine the relationship between haematological parameters and MetS in the adult population of southeastern Iran, Kerman.

**Methods:**

This cross‐sectional study was a sub‐analysis of 1033 subjects who participated in the second phase of the Kerman Coronary Artery Disease Risk Factor Study (KERCADRS). Metabolic syndrome was diagnosed according to Adult Treatment Panel III (ATP III) definition. Pearson correlation coefficient was used to investigate the relationship between haematological parameters with age and components of metabolic syndrome. The role of WBC, neutrophil, lymphocyte and monocyte in predicting metabolic syndrome was evaluated using the receiver operating characteristic (ROC) curve.

**Results:**

White blood cell (WBC) and its subcomponent cells count, red cell distribution width (RDW), monocyte to HDL ratio (MHR) and Neutrophil to HDL ratio (NHR) had a significant positive correlation with the severity of MetS. The cut‐off value of WBC was 6.1 (×10^3^/μL), the sensitivity was 70%, the specificity was 52.9% for females, the cut‐off value of WBC was 6.3 (×10^3^/μL), the sensitivity was 68.2% and the specificity was 46.7%, for males.

**Conclusion:**

WBC and its subcomponent count, RDW, MHR and NHR parameters are valuable biomarkers for further risk appraisal of MetS in adults. These markers are helpful in early diagnoses of individuals with MetS.

## Graphical abstract

Chronic inflammation plays a role in Metabolic syndrome (MetS); hematologic inflammatory parameters can be used as MetS predicting factors.Adults with high WBC count, RDW, MHR, and NHR without any associated underlying chronic disease must be screened because they are at high risk of developing MetS.

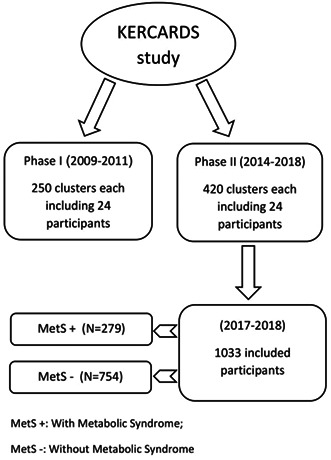

## 1 INTRODUCTION

Metabolic syndrome (MetS) has had different definitions since 1988, which was first introduced by Reaven.^1^ Based on the latest definition of this syndrome, MetS includes at least three factors from the following disorders: central obesity, hypertension, elevated fasting glucose and dyslipidemia (reduced high‐density lipoprotein (HDL) or elevated triglycerides (TG)),^2^ which increases the risk of insulin resistance, diabetes mellitus, cerebrovascular disease, cardiovascular disease, common cancers, osteoporosis and total mortality.^3^, ^4^, ^5^

Prevalence and incidence of MetS have increased significantly following the increase in urbanization, improper nutrition and lack of physical activity, and it has become a global health concern.^6^, ^7^ Although the underlying mechanism of MetS has not been known yet, oxidative stress, chronic inflammation and insulin resistance seem to be the most likely mechanism.^8^

A growing number of studies emphasize the association of MetS components and haematological parameters, including white blood cell (WBC), red blood cell (RBC), platelet (PLT) count and haematocrit (HCT) level as potential indicators markers of thrombotic and inflammatory states.^9^, ^10^, ^11^, ^12^

Meng et al. demonstrated that leukocyte was a good marker for assessing the risk of MetS and cardiovascular disease.^13^ Some studies reported that WBC and PLT counts were significantly correlated with the numbers of MetS components.^14^, ^15^ Ahmadzadeh et al. pointed out that high haemoglobin (HB) levels and HCT can also indicate MetS development.^16^

Since inflammation plays a role in MetS, these haematological inflammatory parameters can be used as MetS predicting factors. Performing cost‐effective CBC tests can easily measure haematological parameters from peripheral blood. Hereditary and environmental factors play an important role in the development of MetS. Currently, no study has investigated the characteristics of MetS and its relationship with blood parameters in the population of southeastern Iran. Therefore, this study aimed to determine the relationship between haematological parameters and MetS in southeastern Iran, Kerman.

## 2 METHODS

### 2.1 Study design and participants

**FIGURE 1:**
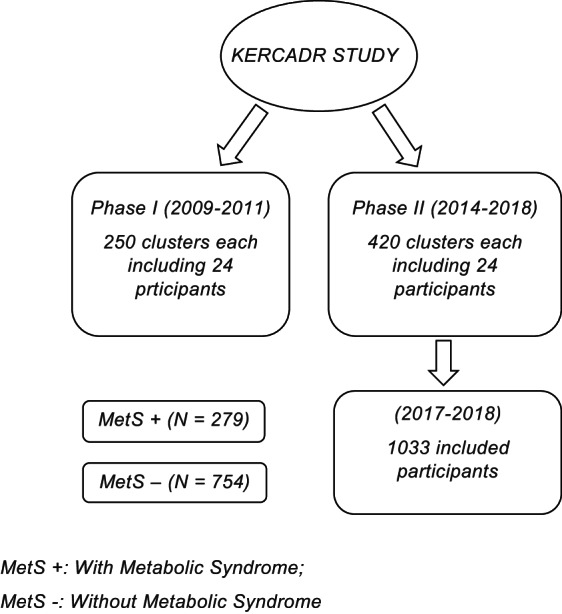
The flowchart of included participants.

This cross‐sectional study was a sub‐analysis of 1033 subjects who participated in the second phase of the Kerman Coronary Artery Disease Risk Factor Study (KERCADRS).^17^ The sampling method was a cluster from the entire population of Kerman residents. In the first phase of the KERCADRS, according to the post‐office list of city residents, 250 postal codes were selected randomly. We invited people over 15 to participate in the study. In the first phase, 24 people were collected in each cluster. In the second phase (420 clusters including 24 participants), people were contacted again, and 1033 who met the inclusion criteria from February 2017 to October 2018 were included in our study (Figure 1). None of the included participants had a history of chronic infectious or inflammatory diseases or the use of any drugs known to affect haematological parameters or lipoprotein metabolism. More details about the data collection method have been published in the study of Najafipour et al.^17^

### 2.2 Data collection

After obtaining informed consent forms from the subjects, demographic data (age, gender) and anthropometric information were collected. A trained interviewer asked participants about cigarette smoking and opium use. People who routinely smoked cigarettes or consumed opium at the time of data collection were considered cigarette smokers and opium addicts, respectively. Height in the standing position without shoes, from heel to head, with an error of 0.5 cm error, weight without shoes and extra clothing with an error of 100 g on a digital scale, body mass index (BMI) which the weight (kg) of the patients was divided by the square of their height (m^2^), waist circumference (WC) in the standing position with 20–30 cm distance between the feet were measured. WC (cm) was measured at the umbilical level. Hip circumference (HC) (cm) was measured based on the largest circumference around the buttocks. Waist‐to‐hip ratio (WHR) was calculated by dividing WC by HC. After 10 min of rest, blood pressure (BP) was measured with a standard manometer from the right arm in the sitting position according to the World Health Organization (WHO) standards, and the blood samples were taken after 12–14 hours of fasting and kept at room temperature. CBC, fasting plasma glucose (FPG) and serum lipids (HDL cholesterol and TG) were tested by routine laboratory methods.

According to Adult Treatment Panel III (ATP III) definition, the presence of at least two of the following five factors is required for the diagnosis of metabolic syndrome: blood pressure over 130/80 mm Hg or consumption of antihypertensive drugs, TG level over 150 mg/dl, FPG over 100 mg/dl or consumption of anti‐diabetic medication like insulin, HDL cholesterol level less than 40 mg/dL (men) or 50 mg/dl (women) and WC over 102 cm (men) or 88 cm (women).

### 2.3 Sample size estimation

In the study of Oda and Kawai, the mean WBC in women with three components of metabolic syndrome was 5416 ± 1163 and in women with only two components of metabolic syndrome was 5077 ± 1358.^18^ The minimum sample size required based on the mentioned numbers and considering the power of 0.8 and alpha of 0.05 for each group was considered to be at least 275 people.

### 2.4 Statistical analysis

Statistical analysis was performed using SPSS version 16 software (SPSS Inc.). Quantitative variables were reported as mean ± standard deviation, and qualitative variables were reported as numbers and percentages. Qualitative variables were compared between the two groups using the Pearson chi‐square or Fisher's exact test. Quantitative variables were compared separately between individuals with and without metabolic syndrome in male and female groups using the independent samples *t* test. Pearson correlation coefficient was used to investigate the relationship between haematological parameters with age and components of metabolic syndrome. The relationship between haematological parameters and the variable number of components of metabolic syndrome was investigated using Spearman's correlation coefficient. The role of WBC, neutrophil, lymphocyte and monocyte in predicting metabolic syndrome was evaluated using the receiver operating characteristic (ROC) curve and with MedCalc® Statistical Software version 20.013 (MedCalc Software Ltd). The optimal cut‐off point was determined using the Youden index.

## 3 RESULTS

A total of 1033 individuals (660 women, 373 men) were included in this study, and the sociodemographic, laboratory parameters and clinical characteristics of the participants are summarized in Table 1.

**TABLE 1:** Basic Characteristics of the Study Subjects.

	Male		*p* value^a^	Female		*p* value^a^
	Normal	Syndrome Metabolic		Normal	Syndrome Metabolic	
	*N* = 291	*N* = 82		*N* = 463	*N* = 197	
Age (years)	45.48 ± 16.21	51.90 ± 13.84	<.001	40.29 ± 14.07	53.33 ± 11.72	<.001
Smoking, *n* (%)	69 (23.7)	18 (22.0)	.739	3 (0.6)	1 (0.5)	.655
Opium addiction, n (%)	64 (22.0)	17 (20.7)	.807	21 (4.5%)	15 (7.6%)	.111
BMI (kg/m2)	24.99 ± 4.22	29.18 ± 3.86	<.001	26.38 ± 4.86	30.81 ± 5.17	<.001
WC (cm)	88.26 ± 11.94	101.29 ± 9.37	<.001	83.30 ± 11.83	98.16 ± 10.47	<.001
WHR	0.89 ± 0.07	0.96 ± 0.05	<.001	0.82 ± 0.08	0.93 ± 0.08	<.001
SBP (mmHg)	114.98 ± 16.01	127.99 ± 16.92	<.001	108.98 ± 15.56	122.60 ± 17.43	<.001
DBP (mmHg)	74.98 ± 9.39	82.38 ± 11.28	<.001	71.79 ± 10.46	78.11 ± 9.46	<.001
FPG	90.86 ± 24.05	116.20 ± 41.39	<.001	86.67 ± 18.34	120.40 ± 47.38	<.001
TG (mg/dl)	127.03 ± 66.25	221.15 ± 163.26	<.001	100.11 ± 43.90	184.71 ± 78.75	<.001
HDL (mg/dl)	46.43 ± 10.55	37.87 ± 7.27	<.001	52.97 ± 12.41	44.31 ± 9.57	<.001
LDL (mg/dl)	109.31 ± 31.35	101.07 ± 37.57	.077	109.29 ± 35.07	113.65 ± 40.42	.188
Cholestrol (mg/dl)	181.08 ± 37.04	181.66 ± 43.56	.912	182.07 ± 43.53	194.74 ± 45.56	.001
WBC (×10^3^/μL)	6.76 ± 1.68	7.08 ± 1.68	.131	6.30 ± 1.66	6.92 ± 1.52	<.001
Neutrophil (×10^3^/μL)	3.46 ± 1.25	3.72 ± 1.38	.119	3.35 ± 1.21	3.70 ± 1.12	.001
Lymphocyte (×10^3^/μL)	2.48 ± 0.73	2.51 ± 0.73	.793	2.24 ± 0.66	2.46 ± 0.68	<.001
Monocyte (×10^3^/μL)	0.58 ± 0.17	0.61 ± 0.16	.174	0.51 ± 0.14	0.54 ± 0.15	.005
RBC (×10^6^/μL)	5.33 ± 0.54	5.35 ± 0.60	.745	4.78 ± 0.47	4.78 ± 0.49	.951
HB (gr/dl)	15.19 ± 1.26	15.33 ± 1.28	.378	13.24 ± 1.18	13.40 ± 1.38	.177
HCT (%)	46.04 ± 3.51	45.87 ± 4.22	.741	41.08 ± 3.57	41.31 ± 4.23	.516
PLT(×10^3^/μL)	220.67 ± 49.95	215.24 ± 45.32	.376	254.18 ± 62.34	255.26 ± 56.46	.834
MPV (fL)	10.27 ± 0.87	10.22 ± 0.77	.640	10.54 ± 0.94	10.47 ± 0.90	.355
RDW‐SD	42.97 ± 3.14	42.76 ± 3.24	.602	42.94 ± 2.98	43.56 ± 3.10	.017
RDW‐CV	13.89 ± 1.35	13.94 ± 1.26	.787	14.02 ± 1.34	14.16 ± 1.39	.242
NLR	1.49 ± 0.68	1.59 ± 0.76	.233	1.57 ± 0.62	1.61 ± 0.67	.539
PLR	94.85 ± 31.90	91.59 ± 28.99	.405	120.76 ± 40.62	110.52 ± 37.39	.003
PMR	403.18 ± 128.29	367.38 ± 96.79	.007	529.20 ± 169.01	499.46 ± 162.80	.037
MHR	0.013 ± 0.005	0.0176 ± 0.01	<.001	0.010 ± 0.004	0.013 ± 0.004	<.001
NHR	0.08 ± 0.04	0.10 ± 0.04	<.001	0.07 ± 0.03	0.09 ± 0.03	<.001

Abbreviations: BMI, Body mass index; DBP, Diastolic blood pressure; FPG, Fasting plasma glucose; HB, Haemoglobin; HCT, Haematocrit; HDL, High‐density lipoprotein; LDL, Low‐density lipoprotein; MHR, Monocyte to HDL ratio; MPV, Mean platelet volume; NHR, Neutrophil to HDL ratio; NLR, Neutrophil to lymphocyte ratio; PLR, Platelet to lymphocyte ratio; PLT, Platelet; PMR, Platelet to monocyte ratio; RBC, Red blood cell; RDW‐CV, Red cell distribution width ‐ coefficient of variation; RDW‐SD, Red cell distribution width ‐ standard deviation; SBP, Systolic blood pressure; TG, Triglycerides; WBC, White blood count; WC, Waist circumference; WHR, Waist‐to‐hip ratio.

^a^ Independent sample t test was used for quantitive variables, and Pearson chi‐square or Fisher's exact test was used for qualitative variables.

In both males and females, in the participants with MetS, age, BMI, WC, WHR, systolic blood pressure (SBP), diastolic blood pressure (DBP), FPG, TG, monocyte to HDL ratio (MHR) and Neutrophil to HDL ratio (NHR) were significantly higher compared with the participants without MetS. In females with MetS, WBC, Red Cell distribution width‐standard deviation (RDW‐SD), Neutrophil, Lymphocyte and Monocyte were significantly higher than the females without MetS (Table 1).

HDL and platelet to Monocyte ratio (PMR) were significantly lower in participants with MetS compared with those without MetS in males and females. In females with MetS, the platelet to Lymphocyte ratio (PLR) was significantly lower than those without MetS (Table 1).

In males with MetS, smoking, opium addiction, WC, WHR, SBP, DBP, monocyte, RBC, HB, HCT, MHR and NHR were significantly higher than these parameters in females with MetS. In females with MetS, BMI, HDL, LDL, cholesterol, PLT, MPV, PLR and PMR were significantly higher than these parameters in males with MetS (Table 2).

**TABLE 2:** Comparison of characteristics between male and female patients with metabolic syndrome.

	Syndrome Metabolic		*p* Value^a^
	Male	Female	
	*N* = 82	*N* = 197	
Age (years)	51.90 ± 13.84	53.33 ± 11.72	0.381
Smoking, *n* (%)	18 (22.0)	1 (0.5)	<0.001
Opium addiction, *n* (%)	17 (20.7)	15 (7.6%)	0.002
BMI (kg/m^2^)	29.18 ± 3.86	30.81 ± 5.17	0.011
WC (cm)	101.29 ± 9.37	98.16 ± 10.47	0.020
WHR	0.96 ± 0.05	0.93 ± 0.08	0.001
SBP (mmHg)	127.99 ± 16.92	122.60 ± 17.43	0.019
DBP (mmHg)	82.38 ± 11.28	78.11 ± 9.46	0.001
FPG	116.20 ± 41.39	120.40 ± 47.38	0.485
TG (mg/dl)	221.15 ± 163.26	184.71 ± 78.75	0.057
HDL (mg/dl)	37.87 ± 7.27	44.31 ± 9.57	<0.001
LDL (mg/dl)	101.07 ± 37.57	113.65 ± 40.42	0.018
Cholestrol (mg/dl)	181.66 ± 43.56	194.74 ± 45.56	0.028
WBC (×10^3^/μL)	7.08 ± 1.68	6.92 ± 1.52	0.434
Neutrophil (×10^3^/μL)	3.72 ± 1.38	3.70 ± 1.12	0.935
Lymphocyte (×10^3^/μL)	2.51 ± 0.73	2.46 ± 0.68	0.640
Monocyte (×10^3^/μL)	0.61 ± 0.16	0.54 ± 0.15	0.001
RBC (×10^6^/μL)	5.35 ± 0.60	4.78 ± 0.49	<0.001
HB (gr/dl)	15.33 ± 1.28	13.40 ± 1.38	<0.001
HCT (%)	45.87 ± 4.22	41.31 ± 4.23	<0.001
PLT(×10^3^/μL)	215.24 ± 45.32	255.26 ± 56.46	<0.001
MPV (fL)	10.22 ± 0.77	10.47 ± 0.90	0.030
RDW‐SD	42.76 ± 3.24	43.56 ± 3.10	0.056
RDW‐CV	13.94 ± 1.26	14.16 ± 1.39	0.212
NLR	1.59 ± 0.76	1.61 ± 0.67	0.883
PLR	91.59 ± 28.99	110.52 ± 37.39	<0.001
PMR	367.38 ± 96.79	499.46 ± 162.80	<0.001
MHR	0.0176 ± 0.01	0.013 ± 0.004	<0.001
NHR	0.10 ± 0.04	0.09 ± 0.03	0.002

Abbreviations: BMI, Body mass index; DBP, Diastolic blood pressure; FPG, Fasting plasma glucose; HB, Haemoglobin; HCT, Haematocrit; HDL, High‐density lipoprotein; LDL, Low‐density lipoprotein; MHR, Monocyte to HDL ratio; MPV, Mean platelet volume; NHR, Neutrophil to HDL ratio; NLR, Neutrophil to lymphocyte ratio; PLR, Platelet to lymphocyte ratio; PLT, Platelet; PMR, Platelet to monocyte ratio; RBC, Red blood cell; RDW‐CV, Red cell distribution width ‐ coefficient of variation; RDW‐SD, Red cell distribution width ‐ standard deviation; SBP, Systolic blood pressure; TG, Triglycerides; WBC, White blood count; WC, Waist circumference; WHR, Waist‐to‐hip ratio.

^a^ The independent sample t test was used for quantitive variables, and the Pearson chi‐square test was used for qualitative variables.

As shown in Table 3, we have considered the number of metabolic components as a measure to determine the severity of MetS, WBC count, Neutrophil, Lymphocyte, Monocyte, RDW‐SD, Red cell distribution width‐coefficient of variation (RDW‐CV), PLR, MHR and NHR parameters that were significantly correlated with the severity of MetS. The correlation was positive in mentioned parameters except for PLR. WBC was significantly correlated with all metabolic components except age (Table 3).

**TABLE 3:** Results of the correlation analysis between the haematological parameters and the components of the metabolic syndrome.

Variables	Age (years)^b^	WC (cm)^b^	FPG (mg/dl)^b^	TG (mg/dl)^b^	HDL (mg/dl)^b^	SBP (mmHg)^b^	DBP (mmHg)^b^	Number of components^a^
WBC (×10^3^/μL)	−0.001	0.139**	0.095**	0.165**	−0.161**	0.080*	0.069*	0.191
Neutrophil (×10^3^/μL)	−0.029	0.122**	0.081**	0.095**	−0.112**	0.049	0.054	0.164**
Lymphocyte (×10^3^/μL)	0.022	0.087**	0.069*	0.188**	−0.138**	0.074*	0.047	0.150**
Monocyte (×10^3^/μL)	0.059	0.113**	0.045	0.089**	−0.139**	0.086**	0.063*	0.089**
RBC (×10^6^/μL)	−0.027	0.097**	−0.021	0.132**	−0.128**	0.154**	0.176**	−0.029
HB (gr/dl)	0.074*	0.130**	−0.001	0.178**	−0.142**	0.150**	0.161**	−0.026
HCT (%)	0.123**	0.136**	−0.044	0.142**	−0.053	0.173**	0.188**	−0.038
PLT (×10^3^/μL)	−0.099**	−0.035	−0.026	−0.005	0.117**	−0.015	0.025	0.034
MPV (fL)	−0.037	−0.031	0.059	−0.085**	−0.038	−0.075*	−0.092**	0.016
RDW‐SD*	0.280**	0.152**	−0.046	−0.026	0.088**	0.080**	0.057	0.067*
RDW‐CV	0.037	0.074*	−0.017	−0.025	−0.033	0.060	0.051	0.106**
NLR	−0.025	0.047	0.030	−0.037	0.002	−0.007	0.020	0.025
PLR	−0.077*	−0.106**	−0.073*	−0.158**	0.195**	−0.074*	−0.022	−0.107**
PMR	−0.101**	−0.126**	−0.059	−0.070*	0.204**	−0.087**	−0.034	−0.054
MHR	0.022	0.231**	0.077*	0.300**	−0.649**	0.080*	0.053	0.335**
NHR	−0.038	0.224**	0.101**	0.268**	−0.579**	0.058	0.054	0.371**

Abbreviations: DBP, Diastolic blood pressure; FPG, Fasting plasma glucose; HB, Haemoglobin; HCT, Haematocrit; HDL, high‐density lipoprotein; MHR, Monocyte to HDL ratio; MPV, Mean platelet volume; NHR, Neutrophil to HDL ratio.NLR, Neutrophil to lymphocyte ratio; PLR, Platelet to lymphocyte ratio; PLT, Platelet; PMR, Platelet to monocyte ratio; RBC, Red blood cell; RDW‐CV, Red cell distribution width ‐ coefficient of variation; RDW‐SD, Red cell distribution width ‐ standard deviation; SBP, Systolic blood pressure; WBC, White blood count; WC, Waist circumference.

*Note*: *p < .05, **p < .01.

^a^ Spearman's correlation coefficient,

^b^ Pearson correlation coefficient.

Genders had different accuracy of WBC, Neutrophil, Lymphocyte and Monocyte in predicting MetS. The accuracy of WBC was higher for females (AUC = 0.632; *p* < .001; 95% confidence interval [CI]: 0.594–0.669) than for males (AUC = 0.564; *p* = .074; 95% confidence interval [CI]: 0.512–0.615) (Table 4).

**TABLE 4:** Areas Under the ROC Curve (AUC), sensitivity and specificity by the optimized cut‐off points for WBC, Neutrophil, Lymphocyte and Monocyte in predicting metabolic syndrome.

	WBC (×10^3^/μL)	Neutrophil (×10^3^/μL)	Lymphocyte (×10^3^/μL)	Monocyte (×10^3^/μL)
Male				
AUC (95% CI)	0.564 (0.512–0.615)	0.566 (0.514–0.617)	0.503 (0.451–0.555)	0.549 (0.497–0.600)
Optimal cut‐off point	6.34	3.41	2.37	0.43
Sensitivity (%)	68.29	57.32	56.10	93.9
Specificity (%)	46.74	56.70	51.89	17.87
Youden index	0.150	0.140	0.080	0.118
*p* Value	.074	.065	.943	.154
Female				
AUC (95% CI)	0.632 (0.594–0.669)	0.608 (0.569–645)	0.609 (0.566–0.642)	0.570 (0.532–0.609)
Optimal cut‐off point	6.15	3.67	2.36	0.44
Sensitivity (%)	70.05	50.25	52.28	77.16
Specificity (%)	52.92	71.0	63.93	36.93
Youden index	0.230	0.212	0.162	0.141
*p* Value	<.001	<.001	<.001	.003

Abbreviation: WBC, White blood count.

**FIGURE 2:**
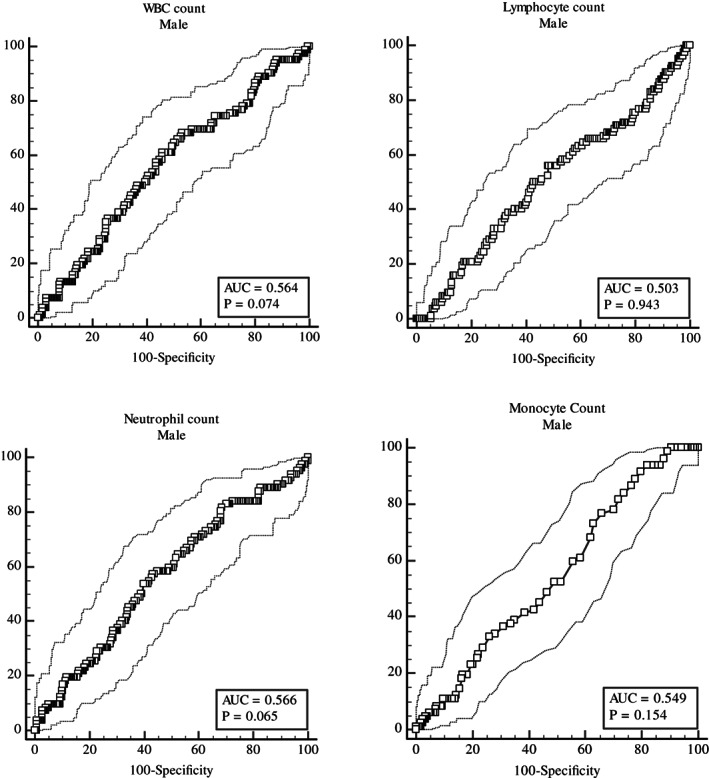
Areas Under the ROC curve (AUC) for WBC, Neutrophil, Lymphocyte and Monocyte in predicting metabolic syndrome for males.

**FIGURE 3:**
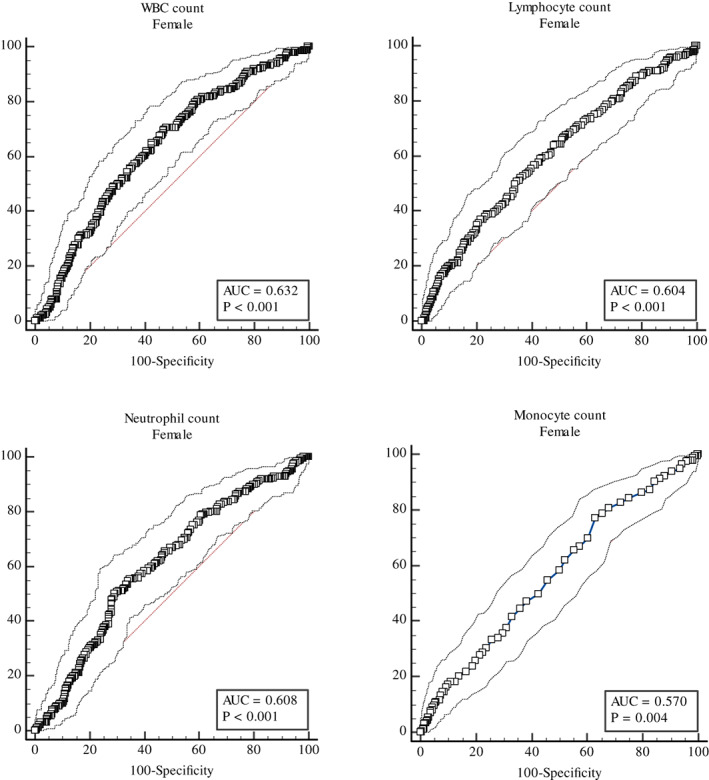
Areas Under the ROC curve (AUC) for WBC, Neutrophil, Lymphocyte and Monocyte in predicting metabolic syndrome for females.

The cut‐off value of WBC was 6.1 (×10^3^/μL), the sensitivity was 70%, the specificity was 52.9% for females, the cut‐off value of WBC was 6.3 (×10^3^/μL), the sensitivity was 68.2%, the specificity was 46.7%, for males. Neutrophil for males (AUC = 0.566) and WBC for females (AUC = 0.632) had better accuracy in predicting MetS compared to other parameters (Table 4) (Figures 2 and 3).

## 4 DISCUSSION

We found that MetS affected the haematological parameters of the patients, including WBC and its subcomponent cell count, RDW, PLR, MHR and NHR.

In our study, WBC and its subcomponent cells count had a significant positive correlation with the severity of MetS, especially in females. Our results were in parallel with previous studies, which had reported a significant difference in the WBC and its subcomponent cells, between participants with or without MetS.^11^, ^16^, ^19^ Yang et al. reported that the number of total leukocyte‐related parameters were elevated in individuals aged 60 years or above.^20^ Ahmadzadeh et al. demonstrated that MetS components were significantly correlated with WBC and its subcomponent cells count.^16^ In Hedayati et al. study in western Iran, the means of WBC count in the MetS group were significantly higher than the control group.^21^ Consistent with our data in a study by Chen et al., contrary to the platelet‐related parameters, the WBC‐related parameters had significant changes in patients with MetS.^22^ In a study on a total of 100 healthy subjects and 200 patients with MetS, total leukocyte and neutrophil counts were significantly increased in all groups of MetS patients compared to the healthy group.^23^

Insulin resistance and chronic inflammation are associated with metabolic syndrome by synthesizing some cytokines leading to an increase in WBC and its subcomponent cells count.^24^ Lorenzo et al. observed an association between the increased risk of diabetes and elevated WBC, neutrophil and lymphocyte counts due to insulin resistance/sensitivity mechanism.^25^ In addition, the relationship between higher levels of WBC count and higher BMI values has been observed in both sexes.^26^

In this study, it was found that in the group of patients with MetS, women had greater BMI, higher cholesterol, PLT and platelet‐related ratios, and men had a history of more smoking and opium consumption, higher BP, HB, HCT, RBC, monocytes and monocytes‐related ratios. Consistent with our results in another study, it has been determined that the predominant feature of MetS in women was abdominal obesity and impaired lipid profile, and in men, it was high BP and impaired lipid profile.^27^

In our study, RDW had a significant positive correlation with the severity of MetS, especially in females; however, this correlation was not observed in mean platelet volume (MPV). So far, minimal studies have been done in this field. Consistent with our data, Farah et al. indicated that both RDW and MPV markers increased as the severity of MetS increased.^28^ Abdel‐Moneim et al. found higher levels of MPV in MetS patients.^23^ Zhao et al. demonstrated that MPV was inversely related to MS in women.^29^ In another study, no significant difference in the MPV between those with and without MetS was observed.^16^

In our study, MHR and NHR had a significant positive correlation with the severity of MetS. A recent study demonstrated that NHR and Lymphocyte to HDL ratio (LHR) were significantly correlated with the prevalence of MetS; also, the correlation was more profound in females.^30^ A recent study showed that both MHR and NHR were significantly increased in patients with nascent MetS.^31^ Considering that monocyte is an indicating factor for inflammatory conditions and atherosclerosis,^32^, ^33^ some studies revealed that the ratio of MHR is a suitable predictor to determine the development and severity of MetS and cardiovascular events.^34^, ^35^

According to our result, Neutrophil to Lymphocyte ratio (NLR) was not recognized as a MetS predictive factor. Ryder et al. observed no association between NLR and obesity or insulin resistance.^36^ Contrary to our results, it was found in two studies that patients with MetS had a higher NLR.^23^, ^37^ Liu et al. relieved that the risk of MetS increased with increasing NLR, and NLR was mentioned as a factor for predicting the development of MetS.^38^ In addition, this ratio has been mentioned as a predictive factor for diabetes in obese individuals.^39^

PLR had a significant negative correlation with all metabolic components except DBP in this study. In another study, it was reported that the amount of PLR in patients with MetS was higher than in patients without MetS, and the amount of PLR had a significant positive correlation with C‐reactive protein (CRP) levels.^40^ In Abdel‐Moneim et al. study, the PLR was significantly higher in all patients with MetS than in healthy subjects.^23^

The cut‐off points for WBC and its subcomponent cell counts are used to determine the potential risk of developing MetS. The cut‐off value of WBC was 6.1 (×10^3^/μL) and 6.3 (×10^3^/μL) for females and males, respectively, in our study. Our results are confirmatory of previous study findings. Pei et al. reported a cut‐off point of 5.6 (×10^3^/μL) for men and 5.8 (×10^3^/μL) for women,^41^ and De Oliveira et al. reported a cut‐off point of 7.5 (×10^3^/μL) for men and 5.6 (×10^3^/μL) for women.^42^

### 4.1 Limitations

Our study had multiple limitations. Firstly, this is a cross‐sectional study, and the analysis of the causative effects was not performed. Secondly, the study sample size was small, and the number of males was smaller than females. Further, our study population included people from southeastern Iran, Kerman; we cannot generalize our results to the whole Iran population.

### 4.2 Future directions

The measurement of haematological parameters is easily available in most parts of the world. However, unfortunately, in public health policies, these parameters do not have a place in the diagnosis and follow‐up of patients with MetS, which will cause these patients to be missed and impose a lot of financial and social costs on the global health system. The results of our study can b

an incentive to conduct prospective studies that will lead to the inclusion of haematological parameters in the diagnostic criteria of MetS. Measuring these haematological parameters is cost‐effective and convenient and facilitates screening patients suspected of MetS and their follow‐up. Prospective studies are required to explain the causality effects between MetS and haematological parameters, confirm our data and evaluate the need to change the risk assessment criteria for MetS.

## 5 CONCLUSION

The higher levels of WBC and its subcomponent cell count, RDW, MHR and NHR could redict an increased chance of developing MetS, regardless of gender differences. WBC also correlated with MetS components, such as WC, FPG, TG, HDL, SBP and DBP; these parameters are easy to access in patients. Considering that no study has been done on this topic in the population of Southeast Iran, our findings provide additional evidence for using these markers for the early detection of MetS components, which ultimately improves the existing clinical practice in identifying and following MetS patients.

## AUTHOR CONTRIBUTIONS

**Mohammad Javad Najafzadeh:** Conceptualization (equal); investigation (equal); writing – original draft (equal); writing – review and editing (equal). **Amir Baniasad:** Conceptualization (equal); formal analysis (equal); methodology (equal); writing – review and editing (equal). **Reza Shahabinejad:** Data curation (equal); investigation (equal); software (equal); writing – original draft (equal). **Mahdieh Mashrooteh:** Formal analysis (equal); investigation (equal); methodology (equal); software (equal). **Dr Hamid Najafipour:** Conceptualization (equal); investigation (equal); methodology (equal); project administration (equal); writing – review and editing (equal). **Mohammad Hossein Gozashti:** Conceptualization (equal); investigation (equal); methodology (equal); project administration (equal); writing – original draft (equal); writing – review and editing (equal).

## FUNDING INFORMATION

The Kerman University of Medical Sciences funded this research project (Reg. No. 95000008).

## CONFLICT OF INTEREST

The authors declare that they have no conflict of interest.

## ETHICAL APPROVAL

The study protocol was reviewed and approved by the ethics committee of the Kerman University of Medical Sciences (ethic code: IR.KMU.REC.1395.775). Informed consent was obtained from all participants in the study.

## Data Availability

The data supporting this study's findings are available from the corresponding author upon reasonable request.
